# 

**Published:** 1919-10-04

**Authors:** 


### THE HOSPITAL

*THE MODERN NEWSPAPER OF ADMINISTRATIVE MEDICINE AND INSTITUTIONAL LIFE*

INCLUDING

ADMINISTRATION, NATIONAL INSURANCE, HEALTH

AND

THE INTERESTS OF ALL INSTITUTIONAL WORKERS

OCTOBER 1919 TO MARCH 1920

VOLUME LXVII

LONDON

PUBLISHED BY THE SCIENTIFIC PRESS (LIMITED), AT *THE HOSPITAL* BUILDING

28 & 29 SOUTHAMPTON STREET, STRAND, W.C. 2.

MDCCCCXX

19362

THE NEW YORK
PUBLIC LIBRARY
CANCELLED
883280
ASTOR, LENOX AND
TILDEN FOUNDATIONS
R 1920 L

### INDEX TO VOL. LXVII.

Abdominal Disease, 581
Accident Cases, A Hardship to, 248, 280
Accidents, Causes of, 172
Acton, Mr. F., 205
Addison, Dr., 36
Administration, The Reconstruction of, 213
Adopted Children, 623
Advertising: A New Channel, 5
Aeroplane Research, 453
Africa, With a Garrison Battalion to, 34
After-War Conditions, 101, 277; Voluntary Hospital Developments, 189
Air, Cleansing the, 621
Alcohol and the Human Body, 207; and Infancy, 482
Alexandra Day, 129, 529
All Souls' and All Saints' Day, 87, 95
Ambulances, Home Service Scheme, 515
America (*see* United States)
American Hospital Association, 464
*American Journal of Nursing*, 565
Anæsthetics, Amateur, 226; Administration of, 232, 246, 300; A New Use for, 395
Anatomy Act Reform, 484, 558
Animal Growth, The Control of, 557
Animals, Sickness Amongst, 88
Anonymous Donor, An, 250
Anthrax Cases, New, 158
Appeal Boards, New Medical, 460
Appeals and Stamped Addressed Envelopes, 281
**APPOINTMENTS, MEDICAL:** 130, 285, 306, 398, 524
**APPOINTMENTS, MATRONS' AND SISTERS':** 22, 44, 64, 108, 130, 152, Nov. 22, vii, 200, Dec. 6, viii, Dec. 13, vi, 260, Jan. 3, vi, 374, 398, 424, Feb. 14, vii, 500, 524, 550, March 13, vi, March 20, vi, March 27, v
Argentina, Hospital Activities in, 82
Armistice Day, 147
Armitage, Mr. C. H., 249
Armstrong-Jones, Sir Robert, 153
Army Nurses (*see also* Demobilisation), Disabled, 58, 62, 148; Uniform Allowance, 58; Bonus, 103; Mesopotamia, 152; Recognition, 495
Artificial Limbs, 47
Assault on a Nurse, 565
Association of Certificated Blind Masseurs, 371, 508
Association of Hospital Matrons, 489, 592
Association of Surgeons, The, 375
Astor, Lady, 203, 217, 543
Atmospheric Pollution, 359
Australia: Nurses' Registration, 28; Bush Nurses, 37, 172; Notes, 197
Auxiliary Services, Uniformed, 107

Baby Week (*see* Infant Welfare)
Bagthorpe Infirmary, 280
Baltic and Lloyds Ambulance Funds, 250
Banbridge Infirmary, 221
Barton, Miss E., 620
Bellevue Training-School for Nurses, 339
Benefactors, Recognition of, 570
Benyon, Major J. W., 48
Bermondsey: New Children's Hospital, 50; Infirmary, 114, 281, 522
Birkenhead Union Infirmary, 466
Birmingham: Relief in Central Europe, 414
Birth-Rate Problem, The, 53, 470
Births: Control, 3; Increase, 249
Blind, Future of the, 55, 72, 144, 188, 364; Blind Masseuses, 104; Grants, 477; The New Bill, 597
Board of Education: Midwives' Training, 372
Bogus Nurse, 390
Bolton, Mr. N. G., 607
Bolton War Hospital, 195
Bona Fide Nurse, 237
Bonsor, Mr. Cosmo, 203
**BOOK WORLD:**
Anon.: Letters from Another Battlefield, 308
Barber-Wells: A Medley of Verse, 308
Berkeley: A Guide to Gynæcology in General Practice, 307; Gynæcology for Nurses, 535
Blackham: Pocket Guide to First-Aid in Poisoning, 308
Brereton: The Great War and the R.A.M.C., 211
Burnett: Manual of Diseases of Children, 308
Cares: The Welfare of the School Child, 535
Cole: Mental Diseases, 435
Coleman: The People's Health, 10
Cooke: Groundwork of Surgery, 307
Cope: Elementary Bandaging and Surgical Dressing, 436
Davies: Surgery of the Lung and Pleura, 98
Dick: Defective Housing and the Growth of Children, 98
Dowd and Jamieson: Food: Its Composition and Preparation, 536
Dunn: The Natural History of the Child, 212
Freeman: Elements of Pediatrics, 10
Gallichan: Letters of a Young Man on Love and Health, 308
Gardner: The Redemption of Religion, 211
Gardner: Handbook of Skin Diseases, 308
Gervis: Arms and the Doctor, 536

Book World—*continued.*
Hill: Food, 10
Incorporated Sanitary Association of Scotland, Transactions, 535
Jack: Wheeler's Handbook of Medicine, 536
Jones: Samuel Butler, 97
Jones: The Road to En-Dor, 435
Keith: Menders of the Maimed, 212
Lamb: The Urethroscope, 9
Laurens: Oto-Rhino Laryngology, 535
Lyndall: Hospital Sketches, 98
McKail: A Public Medical Service, 307
McLaren: A History of the Scottish Women's Hospitals, 536
Macmillan: Cancer Research and Vivisection, 98
McVail: Half a Century of Smallpox and Vaccination, 436
Maghill: Notes on Galvanism and Faradism, 10
Magian: The Practitioner's Manual of Venereal Diseases, 436
Mothercraft, 212
Myer: Lectures on Venereal Disease, 9
Myers: Present-Day Applications of Psychology, 9
Oldfield: Herman's Difficult Labour, 435
Robertson: Housing and the Public Health, 9
Ross: Psychologies, 535
Sargeant: Elementary Organic Chemistry, 536
Schofield: Modern Spiritism, 436
Scurfield: Infant and Young Child Welfare, 10
Smith: Shell-Shock, 10
Stevens: Diseases of Women, 307
Warren: Alcmaeon, Hypermestra, Caeneus, 436
Warrington: School Hygiene, 535
Woollam: Idealus Odsfish, Author, 10
Boot, Sir Jesse, 38
Bott, Major Thomas, 503
Bradford: Municipal Hospital, 327; Starving Children, 467
Braintree Board of Guardians, 541
Brand, Mr. W. J. H., 401, 503
Brentford Guardians, 490
Brighton: Sunday Fund, 371; Club for Nurses, 515
Bristol: Red Cross Grants, 5; Shortage of Nurses, 256
British Federation of Medical Societies, 248
British Hospitals Association, 181, 248, 353, 355, 421
*British Journal of Experimental Pathology, The*, 553

British Medical Association, 281, 357
British Orthopædic Association, 158
British Science Guild, 357
Browne, Dame Sidney, 242, 243, 296, 514, 620
Bruce-Vaughan, Col., 247
Brussels, Health Congress In, 299, 624
Bubonic Plague, 230
Buchanan, Mr. G. S., 427
Buildings Contemplated and in Progress, 127, 199, 397, 486
Burdett, Sir Henry, 60, 527
**BUREAU OF INFORMATION, OUR** (*see* The Institutional Worker—The Supplement to THE HOSPITAL)
Burnett, Sir Napier, 577
Business Habits, The Nurse's Lack of, 255

Camberwell Board of Guardians, 38
Cameron, Sir Charles, 247
Canadian Red Cross Society, 195
Canterbury: St. Augustine's College, 327; Maternity Hostel, 413
Cape Town: Nurses' Salaries, 104
Cardiff: Whitchurch Mental Hospital, 369; Hospital for the Limbless, 478; Mental Hospital, 490
Carlisle: Red Cross Service, 70
Carroll, Mr. F. P., 70
Cavell, Edith, 18, 171, 194, 197, 387, 441, 564; Brussels School, 443; Monument, 564, 589, 592, 618
Census of Health, 517
Central Council for District Nurses, 149, 311, 514, 541
Central Council of Infant and Child Welfare, 471
Central Midwives Board, 16, 60, 196, 238, 542, 620
Chadwick Trustees, The, Dec. 6, xviii, 578
Chaplains, Payment under Poor-Law, 199; Isolation Hospitals, 205
Charity and Commercialism, 551
Charity, The Reconstruction of, 287
Chesser, Dr. E. S., 367
Chelsea Infirmary, 593, 620
Children, Diseases of, 301
Child Labour in London, 471
Child Study, 26
Child Welfare (*see* Infant Welfare)
Chinaman, Affray with a, 159
China: Medical and Nursing Services, 172
Christie, Major W., 503
Christmas Contrasts, 253
Christmas in the Hospitals, 311, 315, 343
Chronic Disease, The State in Relation to, 284; Intestinal Stasis, 611
Church and Social Welfare, 472
Church Missionary Society, 82
Cinema, The: In Obstetrics, 143; Helps Birmingham Hospitals, 250; Children and, 385; Damaged Goods, 458
City Hospitals and Casualty Stations, 451
Cocaine Habit in Bengal, The, 606
**COLLEGE OF NURSING** (*see also* State Registration of Nurses): 36, 123, 168, 173, 238, 352, 367, 388, 398, 479, 513, 539
Annual Meeting, 466
Coat of Arms and Badge, 241, 489, 550
Grants to Midwives, 35
Irish College, 78, 440
Register of Nurses, 171
South African Branch, 256
The Nation's Fund, 81, 311, 412, 441, 442 466, Feb. 14, vii, 467, 488, 496, 525, 541, 565
The Quarterly Bulletin, Jan. 13, vi, 338, 524
LOCAL CENTRES:
Birmingham, 64, Nov. 22, vii, 500; Brighton, 64; Bristol, 338; Dublin, 500; Dundee, Dec. 13, vi; Edinburgh, 130, 152,

COLLEGE OF NURSING—*continued.*
200, Dec. 6, xviii, Feb. 14, vii, 500, March 27, v; Liverpool, 86, 130, Nov. 22, vii, 200, Dec. 6, xviii, 296, 374, 420, 524, March 27, v; London, 44, 108, 130, Jan. 3, vi, 338, 374, 420, Feb. 14, vii, March 20, vi; Manchester, 64, Nov. 22, vii, 443, 500. March 20, vi; Newcastle, 194; Nottingham, 86; Oxford, 200, 524
Colonial Medical Services, 133
Commercial Education, 191
*Common Cause, The*, 291
Compensation Cases, Hospitals and, 159
Comrades of the Great War, 466
Constantinople: Fire at British Hospital, 541
Consumption (*see* Tuberculosis)
Contract Practice, The Future of, 226
Contributions from Patients, 572, 580, 599, 626
Convalescent Home Time Table, A, Supp., Nov. 8, 22, 29
Cook, Sir E. T., 25, 36
Cooper, Sir Astley, 326
Co-ordination Movement, The, 455
Coroner, The, 92; A Criticism, 134; Plain Words, 357
Cost of Disease, The, 205
Cottage Benefit Nursing Association, 107
Cottage Hospital Movement, The, 114
Cotton, The Trade and Hospitals, 555
County Folk Visitation Society, 292
Cowlin, Miss, 540
Cox, Mr. M., 69
Crawford, The Earl of, 429
Crawford, Rev. J. C., 129
Creagh, Mrs., 38
Crime, After-War, 117
Crippled Children, 47
Cripples, A Census of, 158
Crisis, The Hospital, 111, 133, 155, 179, 181, 201, 233, 323, 526, 603, 625
Croydon Poor-Law Infirmary, 291
Crumb of Comfort for Our Croakers, 5
Crumpsall Infirmary, 17
Cumberland and Westmorland Mental Hospital, 420
Cummins, Miss, 541
Cured Patient, The, 84
Curious Case, A, 302
Curry, The Assault on Mr., 178

*Daily News, The*, and Nurses, 59, 82, 565
Dairy Produce, The Question of, 476
Dancing in Relation to the Use of Artificial Legs, 304
Darling, Mr. Justice, 622
Davidson, Sir J. M., Memorial to, 553
Davis, Mr. A. W., 68
Davos Platz, Avalanche at, 311
Dawson, Sir B. E., 325, 429
Daylight Saving, 528
Death Certificates, 314
Demobilisation of Nursing Sisters, 80, 490; Medical Men, 178, 198, 220
Denison House Committee on Public Assistance, 501
Dental: Unqualified Dentists, 152; The Ministry of Health, 614
Dermatology, The New, 137
Devizes Isolation Hospital, 205
Diarrhœa, Chronic, 139
Dickinson, Miss L., 258
Didworthy Sanatorium, The, 61
Diphtheria and Antitoxin, 251, 279, 314
Disabled, Care of the, 186; Hospital Treatment, 220; Nurses, 389, 413, 542, 547, March 13, iv; Productions of, 401; Village Centres, 598; Neurasthenics, 602
Discharged Soldiers and Trades Unions, 518
Dispensing: Drug Industry in France, 26; Shortage of Medicine Bottles, 27; Legal Qualification, 113; Salaries of Pharmacists, 228; Prescriptions and Profiteering, 281; The Future of Pharmacy, 305; Registration, 341; Panel Chemists' Decision, 384
Dissecting Room Difficulties, 357
District Nurses: Cornwall, 413; Durham, 490
Diverticulitis and its Treatment, 559
Divorce Law, 595
Doctor and Increased Production, The, 1
Doctor's Light Car, The, 145
Dogs' Protection Bill, 622
Drugs (*see also* Dispensing), The Week's Market In, 6, 28, 50, 71, 92, 114, 136, 160, 184, 206, 228, 250, 282, 302, 329, 358, 382, 405, 420, 456, 480, 506, 530, 556, 580, 608
Dublin Centralisation Scheme, 327
Dublin Hospitals, Future of, 358
Duchess of Westminster's Hospital, Le Touquet, 389
Dudgeon, Dr. L., 140
Durban, Proposed Hospital, 328
Durham County Council, 114, 443, 520
Dust, 20

Economics of Housing, The, 486
Edinburgh: King Edward Memorial Home, 200
**EDITOR'S LETTER-BOX, THE:**
A Much Under-paid Profession, 521, 547, 571
Bermondsey Hospital, The Staff of, 571
Certified Nurses and Trades Unions, 61
College of Nursing and the Minister of Health, The, 39
Compulsory Registration of Nurses, 313
Corruption of Charity, The, 341
Disabled Nurses, Pensions for, 83
District Nurse and the Measles Campaign, The, 371
Eight-hour Day in Private Nursing, The, 19, 419
Feeble-minded, The, 314
Forty-eight Hours' Bill, 313, 341
Geelong's Thank-offering for Peace, 313
Hospital Library, 127
Ierne, 219
Joint Women's V.A.D. Department, 39
King Edward's Hospital Fund Report, 419
L.C.C.'s Employment of Uncertified Nurses, The, 40
Maesteg and District General Hospital, 39
Massage, Does its Practice Provide a Livelihood? 571
Ministry of Health, Nurses and, 106
Not a Produce Broker, 241
Nurses' Co-operation, The, 419
Nurses Unemployed and Under-paid, 105
Nursing Salaries at Wigan, 547
Problems, Some Nurses', 151
Queen Charlotte's Hospital, 19, 61, 83
Salaries of Hospital Officers, The, 473
Scrapping the Trained Nurse, 628
Society for the Prevention and Relief of Cancer, 127
Staff Conditions in Maternity Hospitals, 19
State Registration, The True Inwardness of, 288
Summer Time, The Adoption of, 571
V.A.D.s, Victimisation of, 20
Viscount Knutsford on Nurses' Homes, 547
West Suffolk Hospital, 495
Wild Women of Mortimer Hall, The, 105
Edmonton District Council, 228
Edmonton Guardians, 328
Egypt: The Travelling Hospitals of, 91
Eight Hours Day (*see also* Forty-eight Hour Week), 58, 215
Electric Lifts for Hospitals, 497
Electro-Medical Diploma, The, 300
Emigration, Women and, 395

Employers' Contributions, 554
Encephalitis Lethargica, 481
Endocrinology, Some Aspects of, 115
England, Wales and Scotland Awakening, 377
*Englishwoman* Exhibition, The, 150
Essex County Council, 60
Evans, Sons, Lescher and Webb, Ltd., 70, 108
Eyes Front, 297
Ex-Service Man, The, 279

Factories, Safety First Movement, 91; Health in, 224, 417
Famine Areas, 494
Fatigue, 17; Effect of Hot Weather on, 162
Federation of Medical and Allied Societies, 477
Feet, The, Mechanical Disabilities, 51
Fenwick, Mr. E. H., 68
Fever Patients, Nursing in Rural Districts, 411
Fever Hospital, Staff Required for a, Supp. Dec. 27
Fevers from the East, 229
Finance, Hospital (*see* Voluntary Hospitals' Finance)
Fisher, Miss L. C., 124
Flanders, Some Landmarks in, 31
Flies and Dysentery, 140
Fletcher, Mr. F., 90, 116
Flying and Air Sickness, 360
FOOD (*see also* Milk): Prosecutions, 4; Dutch Butter, 59; Register of Consumption, 81; Rationing, 129; Release of Meat Supplies, 292; Deaths from Food-Poisoning, 357; Protection, 415; Prices, 519; Serious Aspect, 545
Forthcoming Events, 42, 200, 550, March 13, vi, March 20, vi, March 27, v
Forty-Eight-Hour Week, The, 174, 236, 291, 311, 337, 388, 414, 442, 443
Foster, Miss Janet, 541
Fouracre, Mr. R., 282
France, Drug Industry, 26; Red Cross, 80, 170, 240
Francis, Dr., 506
Franklin, Mr. Phillip, 247
Fraser, Sir Thomas, 357
Fraud on a Nurse, 566
Friends' Emergency Relief Committee, 340
From Across the Seas, 32, 464
Fundus Oculi, The, 117
Furley, Sir John, 3

Garrett-Anderson, Sir Alan, 618
Garrod, Sir A. E., 527
Gas, Is There Hope of Better? 160
General Medical Council, 111, 134, 183
General Nursing Council, 487
Germany, Food Conditions in, 33; People's Losses, 91
GIFTS AND BEQUESTS, 22, 40, Nov. 8, ix, 143, 198, 342, 371, 596, 427
Gilliatt, Miss, 200
Girton College, 389
Glands, The Grafting of, 187
Glasgow, Unique Opportunity, 119, 182; Hospital Outlook, 574
Glassware, Scientific, 453
Gonorrhœa (*see* Venereal Diseases)
Gordon Memorial College, 403
Government and the Voluntary Hospitals, The, 305
Government Debt to Hospitals, 133
Graduate Education, 485
Greenwich Guardians, 390
Griffith, Miss M., 317
Guinea Pig Shortage, A, 326

Hamilton, Lord Claud, 27
Harpur, Mr. C., 248
Hartley, Capt. F., 280
Health and the Rural Districts, 452
Health Organisation in Great Towns, 568
Health Records of 1918, 334
Health, Some Laws of, 14
Health Teaching for the Young, 330
Health Visiting, The Art of, 298
Health Visitors, 26, 49, 516
Health Week, March 27, v
Hemel Hempstead Military Hospital, 314
Hepatic Conditions, Radiography in, 609
Hereford, The Dean of, 69
History Sheets in the Army, Feb. 28, v
Home Office, 81
HONOURS:
Belgian Honours, 102, 218
Greek Honours, 218
Mentioned in Dispatches, 37, 59
Military Medal, 218, 256
New Year, 325
1914 Star, 81
Order of the British Empire, 238, 411
Portuguese Honours, 218
Royal Red Cross, 57, 79, 102, 169, 217, 231, 255, 289, 387, 411, 513, 540, 563, 591, 592, 617
War Medals, 310, 389
Horner, Mr. C. H., Dec. 6, viii
Horseflesh, 138
Horton-Smith, Mr. R. H., 134
HOSPITAL AND INSTITUTIONAL NEWS, 3, 25, 47, 67, 89, 111, 133, 203, 225, 247, 277, 299, 325, 355, 377, 401, 427, 451, 477, 503, 527, 553, 557, 605
HOSPITAL AND INSTITUTIONAL RESULTS, 20, 107, 128, 157, 181, 420, 628
HOSPITAL ARCHITECTURE AND CONSTRUCTION: The New Era, 77, 333, 615
HOSPITALS, INSTITUTIONS, ETC.: (Poor Law, War, and Temporary Hospitals, *see under* individual names.)
METROPOLITAN:
Alexandra Hospital for Children, 430, 552
Bethlem Hospital, 205, Dec. 6, xviii, 542
Bolingbroke Hospital, 280, 302, 599
British Hospital for Women and Babies, 124
Cancer Hospital, 299, 326, 420, 427
Central London Ophthalmic Hospital, 548
Charing Cross Hospital, 605
Chelsea Hospital for Women, 128, 327, 542
City of London Hospital for Diseases of the Chest, 150
City of London Maternity Hospital, 389, 548, 628
Clara Hirsch Convalescent Home, 340
East London Hospital for Children, 348
Evelina Hospital, 300
Foundling Hospital, The, 150
Garrett Anderson Hospital, 136, 249, 602, 618
Great Northern Central Hospital, 17, 59, 64, 68, 70, 129, 259, 321, 401, 410, 515, 542, 564, 572, 626
Guy's Hospital, 20, 44, 129, 189, 203, 228, 250, 279, 315, 317, 345, 433, 449, 453, 461, 584
Hampstead General Hospital, 291, 348
Hospital for Consumption, 222, 368, 467, 510
Hospital for Diseases of the Throat, 134
Hospital for Epilepsy and Paralysis, 489
Hospital for Sick Children, 68, 90, 203, 348, 592
Hospital for Women, 626
House of Charity, Soho, 463
Italian Hospital, 601
King's College Hospital, 130, 249, 320, 548, 570, 599
London Fever Hospital, 107, 401, 453, 503, 531

HOSPITALS, ETC.—*continued.*
London Homœopathic Hospital, Dec. 6, viii
London Hospital, The, 16, 129, 215, 219, 238, 253, 301, 319, 451, 479, 549, 564, 590, 606
London Lock Hospital, 626
London School of Tropical Medicine (*see* Seamen's Hospital)
London Temperance Hospital, 592
Masonic Hospital, 28
Metropolitan Hospital, 128, 522, 606
Middlesex Hospital, 90, 158, 203, 223, 563
Mildmay Mission Hospital, 20, 628
Miller General Hospital, 530
National Children's Home and Orphanage, 64
National Hospital for Diseases of the Heart, 600
National Hospital for the Paralysed and Epileptic, 90, 325, 573, 605, 626
National Institute for the Blind, The, 144
Paddington Green Children's Hospital, 573
Passmore Edwards Hospital, Willesden, 627
Prince of Wales's Hospital, Tottenham, 480, 602
Queen Charlotte's Hospital, 23, 25, 102, 128, 157, March 27, v
Queen Mary's Hospital, Stratford, 218, 222, 403
Queen's Hospital for Children, 129
Royal Dental Hospital, 21
Royal Free Hospital, 36, 449, 540
Royal Hospital for Incurables, Putney, 44, 91, 126, 371, 420, 465, 601
Royal London Ophthalmic Hospital, 128, 548
Royal Lying-In Hospital, 328, 401
Royal Maternity Charity, 128
Royal Surgical Aid Society, 420
Royal Waterloo Hospital, 602
Royal Westminster Ophthalmic Hospital, 573
St. Andrew's Hospital, 628
St. Bartholomew's Hospital, 45, 75, 90, 104, 129, 133, 257, 279, 299, 320, 410, 449, 454, 488, 511, 580, 584, 590
St. George's Hospital, 321, 503, 514, 564
St. Mark's Hospital, 522, 548
St. Mary's Hospital, Plaistow, 548
St. Mary's Hospital, 64, 128, 129, 320, 449, 475, 601
St. Thomas's Hospital, 124, 178, 217, 281, 310, 352, 401, 412, 524, 564
Samaritan Free Hospital, 626
Seamen's Hospital Society, 25, 64, 348, 453, 456, 587
South-Eastern Hospital for Children, 627
South London Hospital for Women, 128
University College Hospital, 428
West End Hospital for Nervous Diseases, 373, 566, 574
Westminster Hospital, 20, 282, 397
West London Hospital, 128, 194
PROVINCIAL, SCOTTISH, AND IRISH:
Aberdare General Hospital, 580
Aberdeen Royal Infirmary, 504
Acton Hospital, 607
Arundel Hospital, 480
Ashton District Infirmary, 555
Barnsley, Beckett Hospital, 503, 527, 572, 579
Bath, Royal United Hospital, 136, 239, 516
Bedford County Hospital, 348
Belfast, Royal Victoria Hospital, 606
Belfast: Irish Hospital for Sick Children, 479
Birkenhead and Wirral Children's Hospital, 506
Birmingham, General Hospital, 135, 326, 385, 489; Queen's Hospital, 385; Princess Alice Orphanage, 412
Blackburn Royal Infirmary, 556, 628

HOSPITALS, ETC.—*continued.*
Bootle Hospital, 69
Bournemouth, Royal Victoria and West Hants Hospital, 28, 280, 382, 556
Bradford Royal Infirmary, 128, 504
Bradley Gale Sanatorium, 454
Brighton, New Sussex Hospital, 5; Royal Sussex Hospital, 135, Nov. 22, vii, 280, 608, 628
Bristol, General Hospital, 20, 239, 292; Royal Infirmary, 478
Bury St. Edmunds, West Suffolk Hospital, 296, 454
Buxton, Devonshire Hospital, 107
Camberley, The Royal Albert Orphanage, 15
Cambridge, Addenbrooke's Hospital, 20
Canterbury, Kent and Canterbury Hospital, 70
Cardiff, King Edward VII. Hospital, 5, 159, 178, 199, 247, 321, 358, 377, 402, 408, 478, 522, 528, 620
Carrickmacross General Hospital, 468
Cheltenham General Hospital, 572
Chester Royal Infirmary, 599
Chingford Memorial Hospital, 64
Clacton Cottage Hospital, 429
Colwyn Bay Cottage Hospital, 206
Coventry and Warwickshire Hospital, 4, 403
Crewe, Memorial Cottage Hospital, 606
Croydon General Hospital, 206, 420
Cumberland Infirmary, 6
Derby Infirmary, 182
Dewsbury General Infirmary, 628
Doncaster Royal Infirmary, 113
Dublin, Richmond, Whitworth, and Hardwick Hospitals, 49, 205; Adelaide Hospital, 489
Dudley, Guest Hospital, 227
Dundee Women's Hospital, 258
Dunfermline and West Fife Hospital, 206
Edinburgh, Royal Infirmary, 107, 128, 405; Longmore Hospital, 222
Exeter, Royal Devon Hospital, 128, 282, 480, 530, 573, 628; Eye Infirmary, 160, 628
Folkestone, Royal Victoria Hospital, 135
Glasgow, Royal Maternity Hospital, 20; Royal Hospital for Sick Children, 20; Western Infirmary, 58, 204, 225, 310; Royal Infirmary, 58, 67, 121, 133, 504, 628; Eye Infirmary, 479
Gloucester Royal Infirmary, 49
Gort, Glenvera Hospital, 594
Gravesend Hospital, 599
Guildford, Royal Surrey County Hospital, 382
Harrow Cottage Hospital, 594
Hastings, East Sussex County Hospital, 292
Huddersfield Royal Infirmary, 555
Ipswich, East Suffolk Hospital, 206, 257, 302, 356, 365, 382
Keighley, Victoria District Hospital, 506, 579
Kidderminster Infirmary, 607
Kingswood, Cossham Memorial Hospital, 37
Leeds General Infirmary, 348, 368, 437, 503
Leicester Infirmary, 85, 133, 152, 348, 499, 522
Leyton War Memorial Hospital, 607
Lincoln County Hospital, 404
Liverpool, Royal Southern Hospital, 38, 128, 368, 369, 592; Children's Hospital, 113; Royal Infirmary, 222, 250, 322, 541, 579; David Lewis Hospital, 579
Maidstone, West Kent General Hospital, 80, 240, 420, 455; Kent County Ophthalmic Hospital, 249
Manchester, Royal Infirmary, 203, 250, 628; Ancoats Hospital, 628

HOSPITALS, ETC.—*continued.*
Margate, Princess Mary's Hospital for Children, 205
Merthyr Hospital, 429
Middlesbrough, North Ormesby Hospital, 627
Midhurst, King Edward VII. Sanatorium, 400
Newcastle-on-Tyne, Royal Victoria Infirmary, 128, 301, 328, 350, 555, 627; City Hospital, 369; Northern Women's Hospital, 555
Newport, Royal Gwent Hospital, 606
Newquay Cottage Hospital, 282
Northampton General Hospital, 157, 350, 548, 628
North Ormesby Hospital, 506
Norwich, Norfolk and Norwich Hospital, 456
Nottingham, Royal Midland Blind Association, 107; General Hospital, 196, 205, 350, 506, 528, 572, 578
Oakham Cottage Hospital, 608
Oxford, Radcliffe Infirmary, 89, 133, 141, 455
Paisley, Royal Alexandra Infirmary, 107
Penzance Hospital, 506
Perth, Murray's Royal Asylum, 420
Pontypool District Hospital, 50, 628
Portsmouth Royal Hospital, 529, 599
Preston Royal Infirmary, 530
Ramsgate General Hospital, 258
Reading, Royal Berkshire Hospital, 350, 600
Redruth Hospital, 280
Reigate Home for Soldiers' Babies, 580
Rhyl, Royal Alexandra Hospital, 594
Rochester, St. Bartholomew's Hospital, 611
Rye, Memorial Hospital, 413
St. Leonards, Eversfield Chest Hospital, 227
Salisbury Infirmary, 351, 573
Sheffield Royal Infirmary, 594
Shrewsbury, Royal Salop Infirmary, 499, 628
Southampton, Children's Hospital, 113
Southport Convalescent Hospital, 530
Stamford Infirmary, 182
Stockport General Infirmary, 351
Stoke-on-Trent, North Staffordshire Infirmary, 379
Stratton Cottage Hospital, 478
Sunderland Royal Infirmary, 600
Tonbridge Cottage Hospital, 488
Torquay, Torbay Hospital, 607
Ventnor, Royal National Consumption Hospital, 107
Wakefield, Clayton Hospital, 607
Walsall Hospital, 281, 573
Walthamstow Dispensary, 607
Warneford Hospital, 430, 456
West Bromwich and District Hospital, 20, 468
Wigan, Royal Albert Edward Infirmary, 6, 205, 352, 505, 515, 574
Winchester, Royal Hants County Hospital, 196, 257, 352, 499
Windsor, King Edward VII. Hospital, 628
Wolverhampton General Hospital, 204, 420, 600
Worcester General Infirmary, 112, 226
Yarrow Convalescent Home, 206
York County Hospital, 171
COLONIAL, U.S.A., &c.:
Adelaide Hospital, 464
Boksburg Hospital, 522
Brisbane, St. Martin's Hospital, 326
Christ Church Hospital, New Zealand, 195
Cape Town, Children's Hospital, 26
Dvinsk, Children's Hospital, 488
Grahamstown, New Albany Hospital, 113
Jerusalem, British Ophthalmic Hospital, 281

HOSPITALS, ETC.—*continued.*
Johannesburg, General Hospital, 191, 199, 522, 555; Children's Hospital, 586
Massachussetts, Newton Hospital, 601
Melbourne, Alfred Hospital, 325; Melbourne Hospital, 339
New York, Flower Hospital, 414
Paris, British Hospital, 136
Pietersmaritzburg, Grey's Hospital, 92, 556
Sydney, Prince Alfred Hospital, 381, 402
Tasmania, Launceston General Hospital, 240; Hobart Hospital, 380
Hospital Journals and Gazettes, The, 129, 590
Hospital Officers' Association, 249
Hospital Saturday Fund, 67, 279, 358, 506; *The Journal*, 554
Hospital Sunday Fund, 295, 455
Hospital Tickets and a Pound of Tea, 227
Hospitals and the Taxpayer, 501
House-to-House Collections, 301
Housing, 25, 63, 396; West Ham, 49; Evictions, 386; Inner London, 402; Hospitals and Housing Schemes, 456
Hove Crèche Fund, Nov. 22, vii
Howell, Mr. F. W., 299
Huddersfield Victoria Nurses' Association, 258
Hutchinson, Dr. Robert, 139

Ideal Home Exhibition, 445, Supp. Feb. 21, 517
"Ierne," A Question for, 69; On Guard, 192
Illegitimacy, 569; Rate Aid for Mothers, 623
Imperial Nurses' Club, 619
"Inasmuch," 106
Income-Tax Liabilities and Claims, 11, 67
Incorporated Society of Trained Masseuses, The, 43
Increased Cost of Living, 368
Increased Doctors' Fees, 226
India, Mortality for 1918, 314; Intemperance in, 505, The Leper Problem, 606
Indian Medical Service, 198, 527, March 27, v
Indian Women as Nurses, 414
Industrial Medicine, 610
INFANT WELFARE AND MATERNITY: (*see also* Midwives and Public Well-being, The): Deptford, 50; St. Pancras, 291; Birmingham, 469; Women's Share, 494; Conference of Scottish Societies, 596; Mortality, 605
Infectious Diseases in London, 198
Infectious Hospital, Cost of Private Case, Supp. Nov. 1; Paying Children Patients, Supp. Feb. 21
Influenza, The Next Wave, 24; Origin of, 68, 111, 130; Research Committees Report, 183, 312; Spraying Contacts, 381; Returning, 428; Precautionary Measures, 549; The Outlook, 553
Inglis, Dr. Elsie, 195
Insane (*see* Mental)
Institute of Hygiene, 416, 472
INSTITUTIONAL FACTS AND FIGURES (*see* The Institutional Worker—the Supp. to the *Hospital*)
Institutional Needs, 397
International Conference of Women Doctors, 17
International Health, Bureau, 491; Work, 624
International Research, 225
International Safeguards for Women and Children, 445
Ipswich Nurses' Home, 240
Ireland, Health Problems in, 286; Nurses' Club in Dublin, 440; Religious and Lay Nursing, 413; Nursing Affairs, 414, 489; Poor-Law Institutions, 490
Irish Nurses' Union, 199
Irish Outrages, The, 227

Irish Public Health Council, 238, 480
Ironworks, In the Surgery of an, Supp. March 13, 1920
Isle of Wight, Health Reform, 50

Jackson Baby, The, 554, 579
Jay, Mr. R. B., 280
Johnson, Mr. C. A., 70
Johnson, Mr. Pussyfoot, 158
Jones, Mr. J., 206
Jones, Sir Robert, 47

Kay, Mr. J., 555
Kelly, Mrs., 60
Kensington Infirmary, 257
Keyser, Miss, 114
Kindness Cure, The, 52
*King Edward*, Rescue of the, 205
King Edward's Hospital Fund for London, 118, 167, 221, 245, 261, 335, 342, 601, 603, 625, March 27, v
King Edward's Fund proposed for Provinces, 328
King George's Fund for Sailors, 342
King's Fund for the Disabled, The, 80
King's National Roll, The, 598
Kingston Board of Buardians, 240, 312, 369
Kitchener Memorial, 478
Knutsford, Lord, 368, 606

Labour, Subscribers and, 403
Lambeth Board of Guardians, 227, 280, 310, 382, 430, 608
Lambeth Infirmary, 248, 310, 478, 591
Lancaster, Mr. A. J., 70
Lancaster-Gaye, Mr. L. L. W., 280
Landon, Dr. 315
Lane, Sir Arbuthnot, 342
Laryngitis, The Severer Forms of, 459, 613
Laundry, The, 368
League of Mercy, The, 293
League of Red Cross Societies, 195
Leeds, Workpeople's Hospital Fund, 49; An Example of Leadership, 437; Trained Nurses' Institution, 468; School Children, 605
Legal Notes for Institutional Workers, 12, 99
Leggett, Mr. J. A., 480
Legislative Advisers, 544
Leicester Health Society, 390
Leicester, V.A.D. Headquarters, 171
Lepers, Missions to, 495; In India, 606
Letter, A Curious, 69
Lewisham Guardians, 171
Life, The Span of Human, 112
Life of a Nurse, 523
Lister Memorial, 119, 380
Literary Medicos, 416
Liverpool Port Sanitary Authority, 7; Lord Mayor's Hospital Appeal, 250; Medical Institution Dinner, 279
Local Government Board, 531
Local Hospitals, The Growth of, 136
London and Counties Medical Protection Society, 554
London and County Rambling Society, 205
London County Council, 6, 135, 194, 290, 336
London County Westminster and Parr's Bank, 402, Feb. 14, vii
London Health Services, 336
London Medical Exhibition, 47
Lost Worker, The, 340
Lucas, J. P., 216
Lumbar Puncture and Late Syphilis, 94
Lyster, Dr. Cecil, 203, 401

Macaulay, The late Col. D., 584
Macdonald, Miss, 196
Macfie, R. C., 52
Mackenzie, Sir James, 399
Macmillan, Miss, 50
McMullen, Mr. W. H., 117
Madness (*see* Mental)
Makins, Sir George, 163, 578
Manchester, 2nd Western General Hospital, 81; Chair of Physiology, 428
Marseilles, British Military Hospital, 130
Massage and Masseuses, L.C.C. Control, 6, 135; Army, 130; Nurses and Massage, 523
Maternity, Peculiar Problem of Beds, 401; Accommodation for Cases, 575; Homes, 519, Feb. 28, v, 583; Increase of Cases in Southwark, 608
**MATRONS' AND SISTERS' DEPARTMENT,** 15, 35, 57, 79, 101, 123, 147, 169, 193, 215, 237, 255, 289, 309, 337, 367, 387, 411, 441, 465, 487, 513, 539, 563, 591, 617
Matrons, Should They be Elected? 512
Matron's Winter Holiday, The, 289
Maw, Mr. Henry, 607
Measles, Notification Withdrawn, 204, 225; Campaign Against, 303
Medical Books, Some Interesting Old, 556
Medical Correspondent, The, 502
Medical Defence Union, 48
Medical Discovery, 325
Medical Education, Some Remarks on, 163
Medical Men, Wills of, 474, 561
Medical Preachers at Manchester, 480
Medico-Psychological Association, The, 99
Meditative Patient, The, 363
Mental, Mortality Among the Insane, 66; Lunacy Law Reform, 99; Nursing, 153; Ex-Soldiers, 178; Ministry of Health, 203; The Trade in Lunatics, 432; Dr. Robertson, 477; Treatment of Incipient Disease, 483; Military Hospitals, Feb. 28, v, 560; Hospital Reform, 580; Report of Board of Control, 585
Mesopotamia, Nursing Services, 220; Demobilisation, 380; R.A.M.C., March 27, v
Metropolitan Asylums Board, 3, 37, 148, 198, 256, 366, 370, 402, 566, 620
Midland Matrons' Association, 444
Midwives (*see also* Public Well-being, The): College of Nursing Grants, 36; Ministry of Health, 59; The Status of the Midwife, 100; Need for Large Training School, 102; Hull Health Committee, 282; Wimborne, 282; Housing, 396
Milestones, 386
Military Cases of Long Standing, 477
Military Furniture and Ambulances, 478
Milk, Adulteration, 4, 25; Shortage, 47; Grading, 448; Dairy Produce, 476; For Babies, 520; Cleansing, 595; Distribution in Manchester, 596; Safe, 604
Millard, Dr. K., 138
Milligan, Sir W., 328
Ministry of Health: Help for Voluntary Hospitals, 5; Advisory Bodies, 15; Housing, 25, 204; Nurses, 56, 122, 338; Midwives, 59; Consultative Councils, 89, 477; Reports, 178; Enham Place, 186; Feeble-minded, 203; Measles, 204; Nurses' Hours, etc., 216, 367; Women Employees, Dec. 13, vi; Organisation, 325; The Blind, 477; Health Information, 491; Medical Referees, 495; Maternity and Child Welfare Centres, 540; Controversy with Lambeth Infirmary, 591; Unqualified Dentists, 614; Nursing Services, March 27, v
Ministry of Labour, 388, 467, 540
Ministry of Pensions, 73, 80, 112, 148, 248, 460, 466
Mitcham, St. John Ambulance, 50
Monmouthshire Nursing Association, 38
Montessori, Dr., 76, 250
Morant, Sir Robert, 577
Morris, Mr. E. W., 451
Mothers' Defence League, 218, 298, 358
Mothers of Disease, 183
Mothers' Pensions, 205, Dec. 13, vi, 299
Motor Invalid Chairs, 549
Motor Show, The, 145
Motor Scooters and Medical Men, 70
Municipal Laboratory, A, 396
Municipal Medical Services, 13
Myles, Sir Thomas, 327

National Association for the Prevention of Tuberculosis, 80, 381
National Council for Combating Venereal Diseases, 360
National Food Reform Association, 104
National Health Insurance: Nursing Scheme, 48, 103; The New Panel Agreements, 156, 391; Failure of, 310, 415; Chemists, 384; Sanatorium Results, 400; Kent Panel Doctors, 402; Doctors Disagree, 448; Amendment Bill, 450; Medical Benefits, 454; The Limited List, 454; Patients Who Discharge Themselves, 505; The Arbitrators Award, 553; New Plans, 598; Medical Remuneration, 602
National Physical Education and Training, 525, 533
National Poor Law Officers' Association, 17
National Relief Fund, 314, March 27, v
National Service, Health Statistics, 545, 567, 597
Native Medicines, Inquiry into, 178
Nervous Disease, Increased Death-rate, 208
Neurasthenic Patient, The Plight of the, 160
Neurological Boards, 527
Newcastle Poor Law Infirmary, 258
New Era in Teaching, The, 2
New South Wales Bush Nursing Association, 256
New Zealand, News from, 32
Nicholay, Mr., Disputed Legacy, 291
Nightingale, Florence, Autograph Letters, 281; Unpublished Letter, 515; Embley Park, 515
Nightingale Fund, The, 59
Northern Nurse, M.D., 18
Norway, Visit to, 239
Not Charity But Humanity, 223
Novel, A Disagreeable, 18
Nurse and the Nation, The 538
Nursery School, The, 418
Nurses' Homes, Baths, 312
Nurses' Co-operation, 149, 241, 338, 372, 473
Nurses' Remuneration and Justice for All, 215
Nurses' Trade Union, The, 101, 104, 146, 149
Nurse, The New, 591
Nurse Who Thinks, The, 15
Nursing and Municipal Economy, 169
Nursing Home, 171

**OBITUARY:** Sir John Furley, 3; Sir E. T. Cook, 25; Mrs. Kelly, 60; Mr. R. H. Horton-Smith, 134; Sir William Osler, 299, 355, 361; Mr. F. W. Howell, 299; Sir Thomas Fraser, 357; Dr. J. Sydney Turner, 380; Dr. Lyster, 401, Mr. W. J. H. Brand, 401; Mr. W. H. Pearce, 410; Major W. Christie, 503; Miss A. L. Pringle, 564; Sir Robert Morant, 577, Col. D. Macaulay, 584
Obstetric Teaching, 509
Occupation, Change of, 159
Old Age and the Thyroid Gland, 71
Ontario Military Hospital, 250
Open-Air Markets and Public Health, 505
Optician, A Nurse's Venture as, Supp. March 27
Orthopædic Hospitals, 314
Osler, Sir William, 299, 355, 361, 527, 561
Out-of-the-Way Institutions, 463
Overworked Nurses, 340

Paddington Guardians, 475
"Pall Mall" Panacea, The, 605
Pantling, Mr. E. K., 70
Paralytic Deformities and Disabilities of the Feet, 93
**PARLIAMENT AND THE PROFESSIONS**, 130, 152, 178, 198, 220, Dec. 13, vi, 314, 341, 494, Feb. 28, v, 549, 601, March 27, v
**PASSING EVENTS AND TOPICS**, 21, 64, 106, 129, 178, 222, 371, 410, 522, 601
Passing Positions of the Moment, 196
Patent Medicines, Profiteering and, 178
Pathologists' Status, The, 158, 204
Pathology, The Future of, 383
Patience, 288
Patterson, Major S. W., 197
Paying Beds, 60, 149, 160
Pearce, Mr. W. H., 410
Pensions for Nurses, 79
People's League of Health, The, 410, 470
Percival, Dr. G. H., 157
Perry, Sir Cooper, 27, 181, 189, 203, 433, 461
Personalia, Nov. 22, vii, 396
Pessimist Again, The, 526
Petrol, The Cost of, 528
Pharmacists (*see* Dispensing)
Physical Education, 157
Physician's Cane, The, 249
Physiology and Medicine, 29
Pituitary Extract, 185
Plague (*see* Bubonic)
Plymouth Maternity Home, 18
Pneumonia, Serum Treatment of, 231
Poland, Typhus in, 82
Police Hospital, 28
Policeman or Doctor? 183
Policewomen, 59, 469
Politics and Hospital Appointments, 478
Poor-Law, Annual Review, 311
Poor-Law Infirmaries, Shortage of Candidates, 36
Ports in Relation to Disease, 7
Poverty and Maternity, Supp., Jan. 24
Preston, Health of, 3; Moor Park Hospital, 414
Preventable Disease, 209
Prevention, The Practice of, 65
Preventive Medicine and the Gymnasium, 405
Prince of Wales, The, 64, 68, 170, 193, 223, 259, 297, 453, 511
Princess Mary, 453, 466, 539
Pringle, Miss A. L., 564
Printers Who Lead the Way, 358
Prisoners of War Fund, 602
Private Nursing, Unpopularity of, Nov. 8, ix, 177
Professional Secrecy, 12
Professional Union of Trained Nurses, 151
Prohibition, 25
Prudential Assurance Company, 555
Psychic Power and Boxing, 463
Psychology, The Nurse's Training, 465; Health and, 493
Psycho-Therapy, 198, 199
Public Health, International, 158; A State View of the Services, 160; Lancashire, 183
Public Pharmacists' Association, 499, 562
Publicity in Villages, 382
**PUBLIC WELL-BEING, THE**: 391, 415, 445, 469, 491, 517, 543, 567, 595, 620

Queen, The: Visit to Hostels, 592
Queen Alexandra, 367, 564, 589
Q.A.I.M.N.S., 103, 238, 444, 490, 549, 593
Q.A.R.N.N.S., 466
Q.M.A.A.C., 59
Queen Mary's Home, Hampstead, 81, 104
Queen Victoria's Jubilee Institute, 238, 338, 466, 592
Quinine, 130

Radiological Research, 553
Railway Strike, 17
Rankin, Dr. Guthrie, 178
Rate-Supported Institutions, The New Nursing Standard, 563
Rats: National Rat Week, 68; Infested Houses, 369
Reading in the Training-School, The Place of, 57
Recent Official Publications, 94, 222, 550
Red Cross: Grants to Bristol, etc., 5; Order of St. John, 16, 42; Peace Arrangements, 27; Grant to Sheffield, 43; Grant to London Hospitals, 221; Florence Nightingale Medal, 149, 301; Work for Voluntary Hospitals, 366, 577; Hereford Branch, 442; Stores and Transport, 443; League of Societies, 444, 517; International Society, 444, 605; Convalescent Home for Nurses, 515; Essex Branch, 542; Cumberland Branch, 566; General Council of Societies, 594, 619; V.A.D. Convalescent Home, 594
Redhill Infirmary, 172
Registrars of Births and Deaths, 607
Research, The Meaning of, 277; Government Help Sought, 527
Roberts, Mr. G. Q., 89, 111
Roberts, Mr. P. N., 49
Rockefeller Institute, 134
Rotherham Borough Council, 136
**ROUND THE HOSPITALS**, 15, 35, 57, 79, 101, 123, 147, 169, 193, 215, 237, 255, 289, 309, 337, 367, 387, 411, 441, 465, 487, 513, 539, 563, 591, 617
Royal Army Medical Corps, 198; Memorial, 553
Royal College of Surgeons, 47, 225, 430
Royal Institute of Public Health, The, 396
Royal National Pension Fund for Nurses, 124, 255
Royal Photographic Society, 319
Royal Sanitary Institute, 81, 90, 129, 183, 371, 470
Royal Society of Medicine, 51, 605
Rugby Final, The Hospitals', 584
Russian Red Cross, 412, 488, 566

St. Andrew's Institute, 399
St. Austell Guardians, 92
St. Dunstan's, 144, 188
St. John of Jerusalem, Order of, 16
Salaries, Sisters' and Nurses': Tynemouth Guardians, 17; Cape Town, 104; Royal Southern Hospital, Liverpool, 369; Prince Alfred Hospital, Sydney, 381; Durham County Council, 443
Sales for Hospitals, 301
Sales of Hospital Equipment, 26
Salford Corporation, 248
Sanatoria and their Critics, 376
*Saturday Fund Journal, The*, 279
Scarborough Nursing Association, 516
Scarlet Fever, 165, 247, 279, 314, 341
Schick Reaction, The, 431
Scholarship for Working-Class Children, 116
School Medical Service, 283, 329
School Monitors, Salaries for, 184
Scotland, Medical Defence in, 111
Scottish Board of Health, 68, 257, 466
Scottish Midwives Association, 60
Scottish Red Cross Hospital, Rouen, 171
Scottish Women's Hospitals, 239
Scrapping the Trained Nurse, 562, 590, 616
Sea-Sickness, 582
Seed Grain, The Electrical-Chemical Treatment of, 30
Serbian Relief, 443
Shanklin Disabled Nurses' Home, 619
Sheffield, Hospital Council, 382; Queen Victoria's Nursing Association, 542
Sheringham Daylight, The, 326
Shirley Warren Infirmary, 620
Shore, Miss F. M., 389, 565
Sibthorp, Mr. C. C., 404
Simpson, Dr. W., 209
Sinclair, Mr. H. A., 566
Sinn Fein and Medical Appointments, 204
Skelling, Miss, 516
Sketches From the Front, 41
Sleeping Sickness, 381
Smaller Medical Fallacies, 532
Small-Pox, 578
Smith, Miss A., 369
Society of Tropical Medicine and Hygiene, 209
Soldiers' Children, The Care of, 24
Soldiers' Tongue, The, 112
South Africa, Nursing Legislation, 413; Trained Nurses' Association, 288; Types of Patients, 556
South American Hospitals, 390, 468
Southwark Board of Guardians, 444, 578
Spain, The Queen of, 194
Sphagnum Moss, 113
Spinal Tumours, Surgical Aspects of, 406
Spiritualism and the Profession, 247
Splint and Artificial Limb Depôt, 467
Sporting Chances, Hospitals and, 504
Stage Children, 417
Starving Continental Children, 516
State Registration of Nurses (*see also* College of Nursing), 147, 148, 171, 175, 193, 214, 216, 220, 256, 290, 309, 314, 337, 339, 387 390
Statistics in Medicine, 552
Statues of Women, 618
Stobhill Hospital, 240
Stoke, Nurses' Hostel, 196
Stratford Nursing Home, 468
Street Collections, 529
Stretford War Memorial, 312
Strike, A Hospital Society and the, 27, 36; Hospital Saturday Fund, 67; Casualties, 113
Students' Rag, The, 158
Surgery of the Front in France, 285
Surgery, The Early Evolution of, 331
Surplus Material, Sales of, 402
Suture Method, A New, 164
Swansea Health Committee, 6
Swift, A New Sidelight on, 184
Sykes, Mr. John, 555

Teaching, Hospital and Medical, 453, 475, 485
Temperamental Selection of Institutional Patients, 306
Test of the Patient, The, 4
Theft from an Operating-Theatre, 506
Thomas, Sir W. J., 247
Thomas Vicary Lecture, The, 225
Thoughts for Nurses, Some, 173
Three Lean Hospital Years Ahead, 233
Tillson, Miss, 49, 86
*Times* Atlas, The, 380
Tobacco Next? 576
Tombolas and their Critics, 74
Training, Facilities in, 35
Tropical Diseases, 112
Tuberculosis: Preston, 3; Financial Aid for Consumptives, 4; Compulsory Removal of Advanced Cases, 8; *Tubercle*, 48; Discharges from Sanatoria, 92; Educative Work Among Nurses, etc., 172; Ex-Service men, 178, 446, 472; Recent Work, 210; Sanatorium Complaints, 236; Ex-Officers, Dec. 13, vi; Sanatoria and Their Critics, 376; Sea Life, 380; National Health Insurance Sanatorium, 400; Welsh National Memorial, 404; Diploma, 454; Birmingham Sanatorium Results, 506; Chemo-Therapy, 510; "Cures," 510; Cheshire Colony, 510; Welsh Insurance Committees, 582; Deptford Scheme, 607

Turner, Dr. J. Sydney, 380
Twilight Sleep, 546
Tynemouth Guardians, 17, 312
Typhus, 48, 390, 457

Unborn, Care of the, 622
Unemployment Insurance for Nurses, 487
Unhealthy Areas, 204
Unidentified Soldiers, 226
Uniform System of Accounts, The, 495, 521
University College of South Wales, 247
United States, Prohibition, 25; Red Cross Relief Unit, 81; American Nurses, 150; Red Cross Commission, 170; Public Health Nurses, 339; Home Helps, 467; Social Work, 492; Care of the Factory Worker, Supp. March 27
Universities and the Nursing Profession, The, 617
Untrained Nurses, 464

Vaccination, Fees, 133; Prophylactic, 184
V.A.D., Social Work, Supp. Oct. 4, 18; Male Workers, 38, 81; Bristol, 82, 238; Future Constitution, 102; Metropolitan Asylums Board, 256; Meetings at Newcastle and Lincoln, 292; Lewisham, 339; Convalescent Home, 594
Valentin, Miss, 36
Venereal Diseases (*see also* National Council), Prevention, 48; Demobilisation, 54; "The End of the Road," 134; The Treatment of Gonococcal Infections, 161; Notification, 225; Arsenic, 300; Sir Arbuthnot Lane, 342; Coroner's Words, 357; Secrecy, 392; Damaged Goods, 458; Syphilis and the Nervous System, 507; Cerebro-Spinal Involvement in Syphilis, 586; Statistics, 602
Victorian Bush Nursing Association, 619
Victoria, Nurse Registration in, 244
Victory Medal (*see* Honours)
Visitors, Regulating a Patient's, Supp. Nov. 29, Dec. 6, 27
Voluntary Help, Utilisation of, Supp. Feb. 14, 21
Voluntary Hospital Finance (*see also under* Crisis), 109, 130, 131, 133, 198, 267, 296, 314, 353, 355, 366, 407, 425
Voluntary System, The Steady Growth of the, 245
Vouvalis, Mr. N. E., 222

Wade, the late Mr. G. T., 326
Wages, Hours, and Prices, 418
Wales, New Maternity Hospital, 48
Walsall Guardians, 82
Walthamstow Health Committee, 228
War, A Lesson of the, 110
War Conditions in the Near East, 434, 537
War Pictures at Burlington House, 409
Warriors' Return, 228
Was Napoleon Responsible? 90
Watson, Mr. James, 368
Way to Win Home, The, 425
Welfare Work, Obstacles to, Supp. Jan. 10; Administration, Supp. Feb. 7
Wellcome Historical Medical Museum, 26
Welsford, Mr. J. H., 194
Welsh Board of Health, 69
Welsh National Memorial, 404
Welsh Nursing Scheme, 516
Welsh Priory, The, 227
West Bromwich Saturday Fund, 228
Westminster Union Infirmary, 369
What is Wrong With Our Hospitals? Supp. Jan. 7, 1920
Which Devour Widows' Houses, 63
Whipps Cross War Hospital, 21
Whitworth Mental Hospital, 258, 340, 619
Wigan, Colliers' Sports, 6
Willesden Urban District Council, 18, 542, 608
Williams, Mr. D., 607
Wilts County Asylum, 420
Wimereux, No. 14 General Hospital, 257, 468
Wise, The Opportunity for the, 123
Woman Workers' World, 41
Women and Children Workers, 418
Women and the State, 543
Women Doctors, 25
Women, Excess of, 469, 493
Women's Auxiliary Services, 58
Women's Holiday Fund, March 20, vi
Women's Industrial League, 470
Woolwich Infirmary, 444
Work and Welfare, 166
Workhouses Disappearing, 479
Working and Learning, 544
Worst War of the World, The, 95

Yell, Mr. W. C., 382
Y.M.C.A. Deficit, 183, 314
York Guardians, 195
Young Man's Opportunity, The, 354

Zoological Interest, A Note With a, 252

PRINTED BY

SPOTTISWOODE, BALLANTYNE AND CO. LTD.

LONDON, COLCHESTER AND ETON

### Hospital and Institutional Results.

**Guy's Hospital.**—The total expenditure of Guy's Hospital now reaches the very important sum of £111,000 a year. If it had not been that 1918 was a particularly good year for legacies it is doubtful whether the balance of income over expenditure, £2,324, could have been shown. The hospital paid in allowances to dependants of employés on active service last year the sum of £2,090.

**Westminster Hospital.**—The annual report has again been considerably abbreviated in consequence of the short supply of paper and for the sake of economy. In answer to a special appeal last year the sum of £11,977, including a gift of £1,000 from the Prince of Wales, who is President, was received. This explains the excess of income over expenditure—viz., £10,717. The hospital balance sheet discloses a mortgage on part of the estate of £23,244.

**Glasgow Royal Maternity & Women's Hospital.**—The almoner's report, which deals largely with the instruction of mothers, is very interesting. Individual teaching in making clothes for infants is given, and cut-out garments ready for making up and "woollies" are sold. Through the kindness of a friend two life-size baby dolls have been provided, and these are beautifully dressed, one in long clothes and one in short clothes, for purposes of instruction. All the garments are made on the simplest and most hygienic lines, and are at the same time pretty and attractive.

**Addenbrooke's Hospital, Cambridge.**—This hospital is very successful in obtaining presents in kind from the country districts surrounding Cambridge, and during 1918 received no fewer than 5,634 fresh eggs. From one village alone were sent 2¾ tons of fruit, potatoes, and vegetables, as well as a substantial gift in money, and in connection with the Hospital Sunday Parade the institution received a large supply of groceries, flour, cleansing material, flannelette, etc.

**Bristol General Hospital.**—The provincial hospitals have recently been very fortunate in not only maintaining but increasing their lists of annual subscribers, and this hospital shows the surprising augmentation of £660 in 1918 as compared with 1917, other headings of income being also increased, and as the expenditure was £4,370 more, the additional help was very useful.

**West Bromwich and District Hospital.**—The expenditure has exceeded the ordinary income by £997, and the accumulated deficiency on the general current account now amounts to £4,582. Certain structural alterations, including better accommodation for the nursing staff, are much needed, but have to be postponed to an indefinite date.

**Royal Hospital for Sick Children, Glasgow.**—The annual report presents a list of requirements, amongst which we notice the equipment of a dietetic kitchen or milk preparation and treatment room. Another want is £40 worth of mercury for a massage apparatus formed out of an 8-inch shell already given. Books for the sisters' and nurses' libraries are always welcome.

**The Mildmay Mission Hospital Bethnal Green.**—The work of the hospital is in the middle of a district that has been much before the public recently. While the visiting staff of doctors has been on active service the Elizabeth Garrett Anderson Hospital has filled the vacant posts.

### DUST.

### Passing Events and Topics.

### Royal Dental Hospital.

The annual dinner of the staff and past and present students of the Royal Dental Hospital is to be revived, and will be held at the Connaught Rooms on Saturday, November 22, at 7 o'clock.

### A War Hospital's Finances.

It speaks well for local interest and enthusiasm to learn that the Social Committee in connection with the Whipps Cross War Hospital will have a surplus of funds to the extent of some £1,400. At a recent meeting it was agreed to distribute this sum by making grants to various funds, among those mentioned for consideration being the Memorial at Leytonstone Parish Church, the Leyton and Leytonstone War Memorial, the Walthamstow Orphans' Country Holiday Fund, St. Dunstan's, and King George's Fund for Sailors.

### MATRONS' AND SISTERS' APPOINTMENTS.

ASHURST WAR HOSPITAL, LITTLEMORE, NEAR OXFORD.—Miss S. L. Mitchell and Miss E. M. Tomlinson have been appointed sisters. Miss Mitchell was trained at St. Luke's Hospital, Halifax, and was afterwards at the Western Fever Hospital, Fulham. She has done private work and military nursing from 1914 until 1919. Miss Tomlinson was trained at the General Hospital, Darlington. She has been sister of the Warneford Mental Hospital, Oxford, and has done military nursing under Q.A.I.M.N.S.R.

BIRMINGHAM AND MIDLAND EYE HOSPITAL.—Miss L. E. Cushon has been appointed matron. She was trained at the London Hospital, and has since been assistant matron at the Lambeth Infirmary and matron of the British Red Cross Hospital, Netley.

BURTON-ON-TRENT GENERAL INFIRMARY. — Miss Ruth Pamplin has been appointed night sister. She was trained at Birmingham General Hospital, and was sister (T.F.N.S.) at the 1st Eastern General Hospital, Cambridge, in Egypt, and in Salonica. Miss Gertrude B. Underwood has been appointed ward sister. She was trained at the Derbyshire Royal Infirmary, where she has since been on the private staff.

CUMBERLAND TUBERCULOSIS COLONY, COTSHILL, NEAR CARLISLE.—Miss Elizabeth Nicholls has been appointed matron. She was trained at the Union Hospital, Sheffield, and has held appointments as inspector of midwives and health visitor (Leicestershire); tuberculosis visitor (Berkshire); matron, Military Families' Hospital, Devonport and Malta; matron, Ministry of Pensions Convalescent Home, Herne Bay; matron, Hull Tuberculosis Colony, Wallington.

DEACONESS HOSPITAL, PLEASANCE, EDINBURGH.—Miss Alice Scruton has been appointed sister. She was trained at the Prince of Wales' General Hospital, Tottenham, and the Hospital for Women, Soho Square, and has since held the posts of ward and theatre sister at Bolingbroke Hospital, London.

DERBYSHIRE ROYAL INFIRMARY, DERBY.—Miss M. G. Allbut has been appointed home sister and sister-tutor. She was trained at the Royal Infirmary, Huddersfield (gold medallist), and has been sister, Clayton Hospital, Wakefield; night sister, sister-housekeeper, and sister-tutor at Queen Charlotte's Hospital, London; military service under the T.F.N.S. Miss M. D. Stafford has been appointed ward sister. She was trained at the Derbyshire Royal Infirmary, and has done district work and military work under the T.F.N.S. The holds the C.M.B. certificate.

EAST SUFFOLK HOSPITAL, IPSWICH.—Miss Janet M. Verity has been appointed sister. She was trained at the Bradford Children's Hospital and Bradford Royal Infirmary, at which latter institution she was afterwards sister. She has also been a staff nurse in Q.A.I.M.N.S.R.

EARBY DISTRICT COUNCIL.—Miss Grace Westwell has been appointed child welfare and health visitor. She was trained at Farnham Infirmary, and has since been at Ilford Council Maternity Home.

GUEST HOSPITAL, DUDLEY.—Miss F. E. Evelyn Harris has been appointed sister. She was trained at St. Bartholomew's Hospital, Rochester, has been sister at the Tiverton Hospital, Devon, the National Hospital for the Paralysed and Epileptic, and at the Hospital for Epilepsy and Paralysis, Maida Vale. Miss Harris holds the certificates of the Incorporated Society of Trained Masseuses and the Central Midwives Board.

HOSPITAL FOR NEURASTHENIA, BRAY COURT, MAIDENHEAD.—Miss Amy Shield has been appointed sister. She was trained at the Royal Bucks Hospital, Reading, and was afterwards engaged in private nursing. She joined the T.F.N.S. in 1914, and saw active service in France.

INFECTIOUS DISEASES HOSPITAL, ASHTON-IN-MAKERFIELD.—Miss Janet Westrap has been reappointed matron. She held this position prior to taking up military duties with the B.E.F. in France.

ISLE OF THANET UNION.—Miss D. Manley has been appointed superintendent nurse. She was trained at the Warrington Union Infirmary, where she was successively ward, night, and home sister. She has since held the temporary post of superintendent nurse at the Isle of Thanet Union and superintendent nurse at the Lincoln Union.

MOLD HOSPITAL, FLINT.—Miss Irene L. Jones has been appointed matron. She was trained at the Bootle Borough Hospital, and has since been matron of the Liverpool Skin Hospital.

MONSALL FEVER HOSPITAL, MANCHESTER.—Misses May Badger, Edith Hadfield, and Dorothy Doig have been appointed sisters. Miss Badger was trained at the Derby Royal Infirmary and at Monsall, and has served in the Q.A.I.M.N.S.R. Miss Hadfield was trained at the Hull Royal Infirmary and at Monsall. Miss Doig was trained at the King Cross Hospital, Dundee, and the Edinburgh Royal Infirmary.

NOTTINGHAM AND MIDLAND EYE INFIRMARY.—Miss F. M. Drakes has been appointed matron. She was trained at Leeds General Infirmary, and has since been sister at Scarborough Hospital, at the Star and Garter Home, Richmond, at the Nottingham and Midland Eye Infirmary, and has done private nursing. Miss Drakes is a member of the College of Nursing. Miss M. Hickey has been appointed sister. She was trained at Manchester Royal Infirmary, where she was later theatre charge nurse. Miss Hickey is a member of the College of Nursing.

NOTTINGHAM GENERAL HOSPITAL.—Miss Margaret Hooper has been appointed home sister. She was trained at the Bolton Infirmary, and has since been staff nurse at the Women and Children's Hospital, Leeds; out-patient, *x*-ray, and ward sister, York County Hospital; home and night sister, Royal Berkshire Hospital, Reading; and assistant matron, Victoria Hospital, Hants.

ROYAL ALEXANDER HOSPITAL FOR SICK CHILDREN, BRIGHTON.—Miss Dorothy Haines has been appointed matron. She was trained at the Poplar Hospital for Accidents, London, and has been ward sister at Manchester Children's Hospital, assistant matron of Liverpool Infirmary for Children, and matron of the Derbyshire Hospital for Children.

ST. MARY'S MATERNITY HOSPITAL, CROYDON.—Miss M. O. Beeham has been appointed sister. She was trained at the General Infirmary, Peterborough, and the Brook Fever Hospital, and she has been sister at Queen Charlotte's Hospital, Bromley Cottage Hospital, and at the National Hospital, Ventnor.

SEVINGTON HOUSE AND PARKFIELD RESIDENTIAL SCHOOLS FOR CRIPPLED CHILDREN, MANCHESTER.—Miss Frances Smith has been appointed matron. She was trained at the General Infirmary, Stockport, and the Seacroft Hospital, Leeds. She has held the posts of sister, District Infirmary, Ashton-under-Lyne; sister, out-patient sister, and night sister Royal Hospital for Sick Children, Edinburgh; and housekeeping sister at the Royal Hospital for Sick Children, Glasgow.

THE INFIRMARY, BECKETT STREET, LEEDS.—Miss Edith M. Shelton has been appointed massage sister and third assistant matron. She was trained at the Edmonton Infirmary, where she was afterwards ward sister. She has been maternity and home sister at the West Bromwich Infirmary; night superintendent and assistant matron, New Zealand Military Hospital, Walton-on-Thames; active service in France and Germany under the Q.A.I.M.N.S. She holds the C.M.B. and massage certificates.

---

SIR ARCHIBALD DAWNAY, Mayor of Wandsworth since 1908, left of his £1 shares in Archibald Dawnay and Sons, Ltd., engineers, 5,000 each to King Edward VII.'s Hospital, Cardiff, and the Putney Hospital; and 500 to the Royal Hospital for Incurables.

### ACCOUNT BOOKS FOR INSTITUTIONS

A Complete Set of Account Books, ruled in accordance with the Uniform System adopted by the King Edward VII. Hospital Fund for London and the Metropolitan Hospital Sunday Fund, especially designed and constructed for the convenience and assistance of Secretaries of Hospitals and all classes of Institutions.

### By Sir HENRY BURDETT, K.C.B., K.C.V.O.

**Series No. 1.—FOR HOSPITALS AND INSTITUTIONS.**

1. ANALYSIS JOURNAL. Royal, 350 pp. Divided into seven sections, with tags.
   Headings as follows:—
   A. Maintenance.
   1. Provisions.
   2. Surgery and Dispensary.
   3. Domestic.
   4. Establishment and Miscellaneous.
   5. Salaries, Wages, etc.
   B. Administration.
   1. Management, Rent, Rates, and Taxes.
   2. Finance.
   C. Extraordinary Expenditure.
   (Uniform with the New Index of Classification adopted by King Edward VII. Hospital Fund for London.)
   Each Heading is ruled with pages containing 15 columns. Price **£3 5s.**
2. CASH BOOK. Foolscap, 300 pp., printed Headings, specially ruled. Price **15s.**
3. CASH ANALYSIS AND RECEIPT BOOK. Foolscap, extra width, 300 pp., ruled with 15 columns, printed Headings identical with Income and Expenditure Table. Price **£1 15s.**
4. SECRETARY'S PETTY CASH BOOK. 300 pp., foolscap ruled with 13 columns, printed Headings identical with Income and Expenditure Table. Price **£1 7s. 6d.**
5. LIST OR REGISTER OF ANNUAL SUBSCRIBERS. Double foolscap, 300 pp., ruled with 10 columns, printed Headings, etc. Price **£2 5s.**
6. ALPHABETICAL REGISTER BOOK. Foolscap, 300 pp., ruled, with date, name and address, and cash columns, cut into alphabetical sections, with totals at end for annual balance. Price **16s.**
7. SUBSCRIPTION REGISTER. Foolscap, ruled with 8 columns, printed Headings, for date when subscriptions become due, name and address, and cash columns. Price **£1 7s. 6d.**
8. LINEN REGISTER. Fcap. long 4to, 300 pp., ruled with columns for Stock, Condemned, Remaining and Issued Linen, also total in Ward and Remarks, also printed Lists of Hospital Linen. Price **12s. 6d.**
9. INCOME AND EXPENDITURE TABLE. For yearly balances and statements. Royal. Price **5d.** per sheet, or **4s.** per dozen.
10. SPECIAL APPEAL ACCOUNT TABLE. Foolscap. Price **2d.** per sheet, or **1s. 6d.** per dozen.

Any of these Books may be purchased separately; a reduction is made if the complete Set is taken. Price of Set complete **£12 net.** Carriage extra.

**Series No. 2.—FOR COTTAGE HOSPITALS AND SMALLER INSTITUTIONS.**

1. CASH ANALYSIS, RECEIPT AND EXPENDITURE BOOK. Double foolscap, 300 pp., ruled with 18 columns, printed Headings identical with Income and Expenditure Table, specially prepared for Cottage Hospitals and Small Institutions. Price **£1 17s. 6d.**
2. SECRETARY'S PETTY CASH BOOK. 300 pp. foolscap, ruled with 13 columns, printed Headings identical with Income and Expenditure Table. Price **£1 7s. 6d.**
3. LIST OR REGISTER OF ANNUAL SUBSCRIBERS. Double foolscap., 300 pp., ruled with 10 columns, printed Headings, etc. Price **£2 5s.**
4. LINEN REGISTER. Foolscap., 300 pp., ruled with columns for Stock, Condemned, Remaining and Issued Linen, also total in Ward and Remarks, also printed Lists of Hospital Linen. Price **12s. 6d.**
5. INCOME AND EXPENDITURE TABLE. For yearly balances and statements. Royal. Price **5d.** per sheet, or **4s.** per dozen.
6. SPECIAL APPEAL ACCOUNT TABLES. Foolscap. Price **2d.** per sheet, or **1s. 6d.** per dozen.

Price of Set complete
**£6 2s. 6d. net.**
Carriage extra.

**The Books are all uniformly bound in half basil, with gilt letterings, and are ruled on best Paper and in every way prepared for practical use at the offices of Institutions.**

PUBLISHED BY
**THE SCIENTIFIC PRESS, LTD.** 28 29 SOUTHAMPTON STREET, STRAND, LONDON, W.C. 2.

